# NADPH Oxidase and Neurodegeneration

**DOI:** 10.2174/157015912804143540

**Published:** 2012-12

**Authors:** Marina S Hernandes, Luiz R G Britto

**Affiliations:** Departamento de Fisiologia e Biofísica, Instituto de Ciências Biomédicas and Núcleo de Apoio à Pesquisa em Neurociência Aplicada, Universidade de São Paulo, SP, Brasil

**Keywords:** Alzheimer´s disease, neuroprotection, Nox enzymes, Parkinson´s disease, reactive oxygen species.

## Abstract

NADPH oxidase (Nox) is a unique, multi-protein, electron transport system that produces large amounts of superoxide *via *the reduction of molecular oxygen. Nox-derived reactive oxygen species (ROS) are known to be involved in a variety of physiological processes, including host defense and signal transduction. However, over the past decade, the involvement of (Nox)-dependent oxidative stress in the pathophysiology of several neurodegenerative diseases has been increasingly recognized. ROS produced by Nox proteins contribute to neurodegenerative diseases through distinct mechanisms, such as oxidation of DNA, proteins, lipids, amino acids and metals, in addition to activation of redox-sensitive signaling pathways. In this review, we discuss the recent literature on Nox involvement in neurodegeneration, focusing on Parkinson and Alzheimer diseases.

## INTRODUCTION

Extensive research over the past few decades indicates that reactive oxygen species (ROS), key mediators of cellular oxidative stress and redox dysregulation, contribute to many pathological events of aging and major disease processes, including cancer [1], cardiovascular disorders [2], diabetes [3] and neurodegenerative diseases [4]. Accordingly, as described in detail in recent reviews [1, 2] a broad range of studies have been developed in animal models and have provided important insight into the involvement of ROS in pathological events in virtually all tissues. Endothelial nitric oxide synthase uncoupling, xanthine oxidase activation, mitochondrial respiration, peroxisome oxidases, cytochrome P-450, among other cellular sources, produce ROS as a byproduct of their biological activity [2]. Indeed, considering the nervous system particular vulnerability to oxidative stress, the expression of NADPH oxidase (Nox) family of superoxide (O_2_^·−^) and hydrogen peroxide (H_2_O_2_)-producing proteins in the brain tissue has been considered unlikely for a long time; however, in the past 15 years Nox family members and the ROS they produce have been implicated in a variety of neurological diseases [5]. This review summarizes current research on Nox involvement in neurodegeneration, with a focus on Parkinson and Alzheimer diseases. 

## REACTIVE OXYGEN SPECIES AND OXIDATIVE STRESS

ROS is a general designation of chemical species arising from oxygen reduction and their related precursors and/or reactive reaction products. These species can be classified into 2 groups of compounds, namely radicals and nonradicals. Superoxide ion radical (O_2_^·−^), hydroxyl radical (OH^.^), peroxyl (ROO^.^) and alkoxyl radicals (RO^.^), and one form of singlet oxygen (^1^O_2_) are among the species classified into the radical group because they contain at least 1 unpaired electron in the shells around the atomic nucleus and are capable of independent existence. The nonradical group of oxygen derivatives contains a large variety of molecules, which include the hypochlorous acid (HClO), ozone (O_3_), hydrogen peroxide (H_2_O_2_) and organic peroxides [6, 7].

A very important notion is the fact that properties such as reactivity, solubility, and diffusibility all make the physiological consequences of each specific ROS very distinct [8, 9]. ROS generation involves a cascade of reactions that generally starts with the production of O_2_^·−^, a molecule with relatively low reaction rates with biological components and unable to cross biological membranes. Oxygen reduction in the presence of a free electron generates O_2_^·−^. Under physiological pH, most of the O_2_^·−^ is in its charged form hydroperoxyl (HO^.^_2_), which has higher reducing capacity, in comparison to O_2_^·−^, and which is able to more easily cross biological membranes. In a hydrophilic environment, both O_2_^·− ^or HO^.^_2_ can reduce ferric ions (Fe^+3^) to ferrous ions (Fe^+2^), enabling them to undergo the Fenton reaction (see later) [7]. 

Superoxide spontaneously dismutate to H_2_O_2_, but this reaction can be catalyzed by superoxide dismutase. H_2_O_2,_ in turn, can easily penetrate biological membranes and react with other species, leading to the generation of more deleterious reactive species, such as OH^. ^or HClO, in the presence of myeloperoxidase. The oxidizing activity of H_2_O_2 _can also lead to deleterious chemical effects such as degradation of haem proteins; iron release; inactivation of enzymes; and oxidation of DNA, lipids, SH groups, and keto acids [5, 7]. However, catalase, glutathione peroxidase, and peroxidases all convert H_2_O_2_ to water and other metabolites within cells, even when H_2_O_2_ is present in very low concentrations [10]. Additionally, Fe^+2 ^interacts with H_2_O_2 _to generate OH^.^ through Fenton’s reaction. The OH^. ^radical is considered to be a powerful oxidizing agent with extremely short life-span that can react at a high rate with organic and inorganic molecules in the cell, including DNA, proteins, lipids, amino acids and metals [10]. 

As the importance of oxidative stress in the pathophysiology of several neurodegenerative diseases is increasingly recognized, an important question is whether cellular ROS generation represents redox signaling as opposed to oxidative imbalance. Under normal conditions, the intracellular redox state, which implies the relative contribution of oxidation and reduction processes to the cell function, is constantly monitored and kept within a narrow range. The redox state plays a significant role in the regulation of signaling pathways, including kinase and phosphatase activity and gene expression through modulation of transcription factor function [e.g., nuclear factor kB (NFkB) and activator protein 1 (AP-1)] [7, 11]. Accumulating experimental evidence demonstrated that protein trafficking, synthesis, degradation and folding are also redox-sensitive processes [12]. The mechanisms responsible for regulating and maintaining the cellular redox homeostasis are not yet fully understood; however, it is widely accepted that alterations in this state toward lower (redosis) or higher (oxidosis) values might lead to several cellular deleterious processes [13, 14]. 

Considering both the toxic and beneficial ROS effects, Helmut Sies [15] defined, in 1985, oxidative stress as “a disturbance in the prooxidant– antioxidant balance in favor of the former”. More recent studies also suggested that alterations of ROS production may be restricted to specific cell compartments such as endosomes, caveolae, nucleus and do not necessarily imply changing the redox status of the major intracellular reductants glutathione or thioredoxin, or even the overall redox state of the cell [16, 17]. 

In addition to this view, the notion that mechanisms of oxidative stress affect several signaling/enzymatic mechanisms and are not limited to free radical damage to macromolecules, provided the basis for the contemporary definition of oxidative stress, as a condition where there is a disruption in the normal function of redox networks with or without free radical-induced macromolecular damage [9, 17].

## NOX FAMILY MEMBERS AND SUBUNITIES

Early Nox research was carried out in polymorphonuclear neutrophils. Since Sbarra and Karnovsky first suggested the existence of such an enzyme in neutrophils, a great deal has been learned about the leukocyte oxidase [18]. Following stimulation, neutrophils undergo a respiratory burst characterized by a 20-fold increase in oxygen consumption, which is accompanied by an increase of glucose utilization and production of reduced NADPH by the pentose phosphate pathway [19]. In parallel with the knowledge produced by understanding the respiratory burst, Nox isoforms have been ascribed an important role in the pathology of cardiovascular disorders such as hypertension and atherosclerosis [20]. On the other hand, despite agreement about the potential importance of Nox enzymes in the pathogenesis of many neurodegenerative diseases, comparatively less is known about mechanisms underlying the regulation of Nox complex activity and expression in brain tissue. 

The NADPH oxidase is a multi-subunit enzyme that transfers electrons across biological membranes. The subunits are localized both in the cell membrane (cytochrome b558, comprised of p22^phox^ and gp91^phox^) and in the cytoplasm (p40^phox^, p47^phox^, and p67^phox^) [21]. Upon stimulation, activation of a low-molecular weight G protein (Rac1 or Rac2) and phosphorylation of p47^phox^ initiates migration of the cytoplasmic elements to the plasma membrane [22]. The catalytic component gp91^phox^ facilitates electron transfer. The electron from cytoplasmic NADPH travels first to flavin adenine dinucleotide (FAD), then through the Nox heme groups, and finally across the membrane and it is transferred to oxygen. Superoxide is the primary product of the electron transfer, but other downstream ROS can also be generated [5]. Seven Nox isoforms have been identified so far: Nox1, gp91^phox^ (Nox2), Nox3, Nox4, Nox5, and Dual Oxidase 1 and 2 (Duox1 and Duox2). Nox 1–5 are known to produce O_2_^·−^, whereas Duox enzymes are able to release H_2_O_2 _without forming a detectable amount of O_2_^·−^, because they contain an extracellular peroxidase-like domain in addition to the EF-hand Ca2+ binding domains and gp91^phox^ homology domain [23]. However, as elegantly demonstrated by Serrander and col. [24], in cell lines expressing Nox4 the type of ROS released was predominantly H_2_O_2_, whereas O_2_^·−^ was almost undetectable. Although close structural and functional similarities exist between the different Nox homologues, each isoform seems to be differentially expressed and regulated across distinct tissue and cell types [25]. Specifically in the central nervous system, the presence of Nox1, Nox2, Nox3, and Nox4 isoforms has been identified in several structures (Table **1**) [26]. However, very little is known about the role of Nox5 and Duox1 and 2 in the nervous tissue. Detailed mechanisms of activation for individual Nox enzymes described in the nervous system are discussed below.

Nox isoforms have distinct activation mechanisms. Nox1 associates with the membrane subunit p22^phox^, and mostly with the cytosolic subunits, NoxO1 (p47^phox^ homologue) and NoxA1 (p67^phox^ homologue) and Rac [27, 28]. Nox2 interacts with p22^phox^, phosphorylated p47^phox^, p67^phox^, and Rac. Nox2 has also been found to complex with p40^phox^, but the functional consequences of this interaction are not clear [2]. Nox3 activation is less well defined, but it is believed to be similar to Nox1, involving Rac, p47^phox^, and NoxA1. Nox4 is unique among the catalytic Nox subunits in that it only interacts with p22^phox^, and it is thought to be constitutively active [2]. Nox1 mRNA was found to be expressed in neurons, astrocytes and microglia, whereas Nox4 was found in neurons. In activated microglia, ROS production is frequently associated with Nox2 expression. However, Nox1 and Nox4 might also play a role in that process [2]. 

## NOX AND PARKINSON’S DISEASE

Parkinson’s disease (PD) is a neurodegenerative disorder characterized by a progressive loss of dopaminergic neurons in the nigrostriatal pathway of the brain, which triggers complex functional modifications within the basal ganglia circuitry, leading primarily to motor dysfunctions. Although the etiology of PD is unknown, a common element of most theories is the involvement of oxidative stress, either as a primary or secondary event of the disease [4, 29-31]. Research on the pathogenesis of PD suggested that mitochondrial dysfunction is the major source of oxidative stress in this disease [32]; however, increasing evidence has been also found for a role of Nox enzymes in the process.

The research on the mechanisms involved in PD disease has relied on the development of animal models that reproduce the pathological and behavioral characteristics of the disease. Classically, these models are based on the systemic or intracerebral administration of neurotoxins capable of selectively degenerate the nigrostriatal system. A very useful model is based on systemic administration or striatal injection of 1-methyl-4-phenyl-1,2,3,6-tetrahydro-pyridine (MPTP), which causes a PD-like syndrome highly similar to the human disease [33-35]. As demonstrated under *in vivo* conditions, translocation of p67^phox^ was induced by MPTP in mouse brain and prevented by the tetracycline derivative minocycline [36]. More recently, it has been demonstrated that p47^phox^ phosphorylation and p47^phox^–gp91^phox^ complexes are significantly increased in mice substantia nigra (SN) after systemic injections of MPTP [37]. In addition, MPTP induced increases of both gp91^phox^ and 3-nitrotyrosine in the SN of ageing mice, which were inhibited by oral treatment with the NO-donating derivative of flurbiprofen [2-fluoro-α-methyl (1,1'-biphenyl)-4-acetic-4-(nitrooxy)butyl ester (HCT1026)] [38]. In line with these findings, degeneration of dopaminergic neurons induced by MPTP was attenuated in gp91^phox-/- ^mice as compared to Wt littermates [39]. In the same PD model, gp91^phox^ immunoreactivity colocalizes with microglial cell markers but not with astrocyte markers, confirming a microglial origin for Nox [40]. *In vitro* overnight MPP+ (a MPTP metabolite) treatment of N27 rat dopaminergic cells was able to induce Nox2 protein expression and O_2_^·−^ generation, as measured by flow cytometric detection. This effect was inhibited by siRNA silencing of p22^phox^ [41]. Thus, it appears that activation of Nox2 plays a relevant role in the loss of dopaminergic neurons in the MPTP-induced PD model. 

Another commonly used procedure for obtaining experimental nigrostriatal lesion in rodents is based on local infusion of 6-hydroxydopamine (6-OHDA) (reviewed in [42]). The biological effects of 6-OHDA are mainly related to the massive oxidative stress caused by the toxin that, once accumulated in the cytosol, seems to be auto-oxidated, promoting a high rate of free radical generation [43]. As detected by dihydroethidium fluorescence, the treatment of primary mesencephalic cultures with 6-OHDA induced a significant increase of the intracellular generation of O_2_^·−^ in dopaminergic neurons, as well as in microglial cells [44]. There is evidence to implicate Nox-derived ROS in this process, but the mechanisms involved are poorly understood. Important advances in this regard were provided by a series of recent studies. For instance, data from our laboratory suggest a relevant role for Nox2 in 6-OHDA-induced PD. In this study, the membrane protein levels of p67^phox^ were markedly elevated in the SN of 6-OHDA lesioned mice, suggesting that the p67^phox^ subunit translocated from the cytosol to the plasma membrane, thus forming a Nox entity capable of producing superoxide after 6-OHDA injection. Tyrosine hydroxylase immunolabeling indicated that gp91^phox-/-^ mice appear to be protected from dopaminergic cell loss in the SN and from dopaminergic terminal loss in the striatum. Moreover, wild type mice treated with apocynin, a Nox inhibitor [45], and gp91^phox-/- ^mice all exhibited significantly ameliorated apomorphine-induced rotational behavior after 6-OHDA lesion (Hernandes *et al*., submitted). These results are corroborated by some *in vitro* observations. In rat primary mesencephalic cultures, 6-OHDA induced a significant increase of gp91^phox^ and p47^phox^ immunolabeling. Confocal microscopy revealed that both gp91^phox^ and p47^phox^ were intensely expressed in microglia cells. Microglial activation and O_2_^·− ^generation in dopaminergic neurons were significantly reduced by apocynin [46]. Six-OHDA also induced increased expression of gp91^phox^ in human dopaminergic neuroblastoma cells [47]. In addition, it has been recently reported that striatal injection of 6-OHDA increased Nox1 expression in dopaminergic neurons of the rat SN. Rac1, a key regulator in the Nox1 system, was also activated. Nox1 was localized into the nucleus, and immunostaining for a DNA oxidative stress marker, 8-oxo-dG, was increased. Adeno-associated virus-mediated Nox1 knockdown and Rac1 inhibition were both able to reduce 6-OHDA-induced oxidative DNA damage and dopaminergic neuronal degeneration [48].

Nevertheless, the role of Nox enzymes in 6-OHDA-induced PD might not be only limited to the Nox2 isoform. As recently demonstrated, striatal administration of 6-OHDA increased Nox1 expression in dopaminergic neurons of the SN. Furthermore, adeno-associated virus-mediated Nox1 knockdown reduced 6-OHDA-induced oxidative DNA damage and dopaminergic neuronal degeneration in the rat SN [48].

The involvement of Nox in PD has also been revealed through other, unrelated PD models. In mesencephalic primary cultures, activated microglia generated Noxderived superoxide and enhanced lipopolysaccharide-elicited dopaminergic neurodegeneration [49]. Furthermore, microglial Nox but not neuronal Nox, renders dopaminergic neurons more sensitive to rotenone, an herbicide able to reproduce features of PD in rats [50]. In mesencephalic neuron-glia cultures from gp91^phox-/-^ mice the deleterious effect of microglia induced by substance P on tyrosine hydroxylase-positive neurons was significantly attenuated [51]. The Nox involvement in the cytotoxic action of paraquat, another widely used parkinsonism inducing agent, has also been recently described. Apocynin attenuated paraquat-induced dopaminergic degeneration, Nox activation, cytochrome c release and caspases-9/-3 and microglia activation. According to the authors, paraquat induces oxidative stress through Nox activation and depletion of glutathione, which in turn activate the apoptotic machinery leading to dopaminergic neurodegeneration [52]. It has been also reported that the Nox inhibitor diphenyleneiodonium (DPI) blocked the paraquat-induced ROS production and subsequent dopaminergic neurodegeneration [53].

## NOX AND ALZHEIMER’S DISEASE

Alzheimer’s disease (AD) is characterized by an initial mild cognitive impairment that progressively develops into a loss of higher cognitive functions, resulting in dementia. Accumulation of amyloid-β peptide (Aβ) in the brain is considered one of the main pathological features of AD. Other AD microscopic hallmarks include abnormal protein folding, exacerbated activation of glial cells, and synaptic and neuronal loss [54]. Importantly, a growing body of evidence supports a role for abnormal Nox activation in this pathology. Activation of Nox2 in the brain of AD subjects has been demonstrated, as evaluated by the translocation of Nox2 subunits [55]. In addition, analysis of frontal lobe tissue of AD patients demonstrated signiﬁcantly increased levels of Nox1 and Nox3 mRNAs, suggesting that other isoforms beyond Nox2 can contribute to that neuropathology [56]. There is also evidence that Nox-associated redox pathways might participate in the early pathogenesis of AD. By using a luminescent assay to detect Nox-dependent ROS production, it was shown that Nox activity is increased in the superior/middle temporal gyri over control levels at the earliest clinical manifestations of disease, but not in late-stage AD. The observed increases of Nox activity were associated with increased expression of p47^phox^ and gp91^phox^ in both microglia and neurons [57].

However, *in vitro* and *in vivo* studies have generated most of the information related to the role of Nox enzymes in AD pathogenesis. It has been recently shown that the cholesterol oxidation product, 24-hydroxycholesterol, markedly potentiates the pro-apoptotic and pro-necrogenic effects of Aβ. This effect depends on its strong enhancement of the intracellular generation of Nox-derived ROS, mainly H_2_O_2_, and the consequent impairment of the neuronal redox state, measured in terms of the GSSG/GSH ratio [58]. Gp91ds-tat, a Nox2 inhibitory peptide, decreased both oxidative stress and AD pathology in aged mice [59]. Also, It has been recently shown that feeding AβPP/PS1 double transgenic mice, a mouse model of AD, with a diet containing phenolic antioxidant tert-butylhydroquinone, inhibits Nox2 protein expression and suppressed lipid peroxidation in the cerebral cortex and hippocampus [60]. It was also found that age-dependent increases of Aβ had a significant linear relationship with both Nox4 activity and cognitive performance in “humanized” APP×PS1 knock-in mice [61]. Apocynin treatment reduces Aβ deposition and the number of microglial cells in the cortex and hippocampus of aged transgenic mice overexpressing the human amyloid precursor protein (hAPP (751)(SL), but it failed to inhibit cytosolic p67^PHOX^ translocation to the membrane and to reduce the levels of TNFα [62]. Similarly, apocynin did not improve cognitive and synaptic deficits, and did not decrease Aβ deposition, microgliosis and hyperphosphorylated tau in transgenic AD mice [63]. *In vitro* exposure of hippocampal neuronal/glial co-cultures to Aβ peptides resulted in activation of glial Nox, followed by neurodegeneration [64]. In another *in vitro* study using a co-culture of microglia and neuroblastoma cells over-expressing the Aβ precursor protein (APP), ROS generated by microglia induced neurodegeneration. This effect was attenuated by ROS-scavengers and was dose-dependently inhibited by DPI, suggesting that APP-dependent microglia activation and subsequent ROS generation by Nox play a crucial role in neuronal degeneration [65]. In addition, some studies demontrated that Aβ-induced Nox2 activation in astrocytes contributes to neurodegeneration [66, 67]. Aside from its involvement in neurodegeneration, production of H_2_O_2_ from Nox2 regulated microglial proliferation induced by Aβ, as demonstrated in a primary mixed glial culture obtained from rat cerebral cortex. This effect was prevented by apocynin and catalase, a H_2_O_2_-degrading enzyme [68]. In summary, strong evidence indicates that oxidative stress in AD involves ROS generation by Nox enzymes, in particular Nox2.

## CONCLUSIONS

Regardless of the general agreement on the potential importance of ROS-generating Nox enzymes in the pathogenesis of many neurodegenerative diseases, our knowledge on the specific molecular mechanisms of activation and subsequent functional consequences of activating specific Nox enzymes in the brain tissue is limited. In addition to examining the local expression and activation of Nox enzymes under neurodegenerative conditions, the cellular consequences of a chronically dysregulated oxidative environment must also be taken into account. For instance, it is necessary to evaluate the ROS-dependent activation of inducible transcription factors and modulation of gene expression. Indeed, as nonspecific scavenging may prevent ROS from acting in essential biochemical pathways, that knowledge is essential to allow and improve the development of specific novel therapies targeting Nox proteins, therefore reducing the pathological consequences of oxidative stress.

## GRANT INFO

Work in the author’s laboratory is supported by grants from FAPESP (Fundação de Amparo à Pesquisa do Estado de São Paulo), CAPES (Coordenação de Aperfeiçoamento de Pessoal de Ensino Superior), University of São Paulo-Núcleo de Apoio a Pesquisa em Neurociência Aplicada - and CNPq (Conselho Nacional de Desenvolvimento Científico e Tecnológico). M.S.H. is the recipient of a fellowship from FAPESP.

## Figures and Tables

**Fig. (1):**
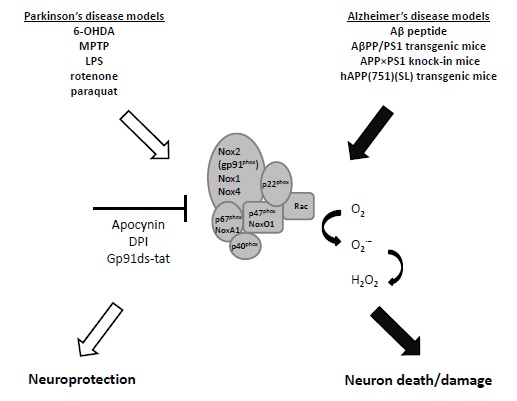
Nox activation, neurodegeneration and neuroprotection. In Parkinson´s and Alzheimer´s disease rodent models, increased activation of the Nox family of ROS-producing proteins contributes to neurodegeneration. Blockade of Nox generates neuroprotection in some instances. Abbreviations: 6-hydroxydopamine (6-OHDA); 1-Methyl-4-Phenyl-1,2,3,6-Tetrahydropyridine (MPTP); Lipopolysaccharide (LPS); amyloid-β peptide (Aβ); superoxide (O_2_^·−^); hydrogen peroxide (H_2_O_2_) and diphenyleneiodonium (DPI).

**Table 1.** NADPH Oxidases in the Brain

	Brain Region	Specie	RNA	Protein	References
**Nox1**	Medulla	Human		+	[85]
	Superior colliculus	Rat	+		[26]
	Hippocampus	Rat	+		[84]
	Cerebellum	Rat	+		[83]
	Forebrain	Mice	+		[25]
	Midbrain	Mice	+		[25]
	Hindbrain	Mice	+		[25]
	Dorsal root ganglion	Mice	+		[86]
	Hypothalamus	Mice	+		[87]
**Nox2**	Medulla	Human/Rat		+	[74,83]
	Superior colliculus	Rat	+		[26]
	Thalamus	Mice/Rat	+	+	[26, 78]
	Hippocampus	Mice/Rat/Human	+	+	[58, 69, 70, 88, 90]
	Cerebellum	Mice		+	[80]
	Forebrain	Mice	+		[25]
	Midbrain	Mice	+		[25]
	Hindbrain	Mice	+		[25]
	Hypothalamus	Mice/Rat	+	+	[73, 74, 76]
	Substantia nigra	Mice/Rat	+	+	[ 75, 77]
	Amygdala	Mice		+	[78]
	Nucleus of the solitary tract	Rat		+	[79]
	Striatum	Mice/Rat	+	+	[71, 72,75]
	Cortex	Mice/Rat/Human	+	+	[24, 69, 89, 90]
	Brainstem	Mice		+	[70]
**Nox3**	Cerebellum	Rat	+		[81, 83]
	Hypothalamus	Rat	+		[81]
	Cortex	Rat	+		[81]
**Nox4**	Superior colliculus	Rat	+		[26]
	Hypothalamus	Mice		+	[73]
	Cortex	Mice	+	+	[61, 82]
	Forebrain	Mice	+		[25]
	Midbrain	Mice	+		[25]
	Hindbrain	Mice	+		[25]
	Hippocampus	Mice	+	+	[82]
	Cerebellum	Mice	+	+	[82]

Observation: several studies that reported Nox protein occurrence by immunostaining have not actually used the appropriate negative controls, and therefore the data must be interpreted with caution.
